# Duration of early empirical antibiotic exposure in very low birth weight infants with suspected early-onset sepsis and associated factors: a retrospective cohort study

**DOI:** 10.3389/fped.2026.1811714

**Published:** 2026-04-29

**Authors:** Catalina Morales-Betancourt, Maria Dolores Canales-Siguero, Marta Fernández-Gaitán, Adriana Montealegre-Pomar, Elena Bergon-Sedín, Concepción De Alba-Romero, Carmen Rosa Pallás-Alonso, Maria Teresa Moral-Pumarega

**Affiliations:** 1 Research Institute Hospital 12 de Octubre, Madrid, Spain; 2 Departament of Neonatology, Hospital Universitario 12 de Octubre, Madrid, Spain; 3 Escuela de Doctorado, Universidad de Alcalá, Ciencias de la salud, Madrid, Spain; 4 Departament of Pharmacy, Hospital Universitario 12 de Octubre, Madrid, Spain; 5 Hospital Universitario San Ignacio, Bogotá, Colombia

**Keywords:** antibiotic exposure, antibiotic stewardship, early-onset sepsis, sepsis, very low birth weight infant

## Abstract

**Introduction:**

Early antibiotic exposure in very low birth weight (VLBW) infants has been linked to alterations in gut microbiota and increased neonatal morbidity. However, data on actual treatment duration and associated factors remain limited.

**Objective:**

To determine antibiotic exposure time, measured in hours of life, in VLBW infants empirically treated for early-onset sepsis (EOS) with negative blood cultures, and to identify factors associated with longer exposure.

**Methods:**

A retrospective cohort study was conducted in a level IIIC neonatal intensive care unit between March 2021 and December 2023. VLBW infants who received empirical antibiotics for EOS risk and had negative blood cultures were included. Clinical, perinatal, and time-related variables were analyzed.

**Results:**

Of 286 admitted VLBW infants, 161 met inclusion criteria. Empirical antibiotics were administered to 56.3% without proven infection. Median time from birth to antibiotic discontinuation order was 45 h for both ampicillin and gentamicin, and 42 h when considering blood culture incubation time. Initiation of antibiotics during weekends was associated with 0.46 additional days of therapy [CI (0.148, 0.762); *p* = 0.004]. Over the study period, antibiotic discontinuation occurred progressively earlier.

**Conclusions:**

Although antibiotic discontinuation is increasingly prompt, VLBW infants remain exposed closer to 48 than to 36 h of life. Being born on weekends is associated with prolonged antibiotic exposure. Awareness of actual exposure times can guide further optimization of antibiotic stewardship in this high-risk population.

## Introduction

Early life exposure to antibiotics has been related with an increased risk of adverse events, especially in very low birth weight infants (VLBW), the exposure to antibiotics without proven infection in the first days of life, has been associated with increased risk of bronchopulmonary dysplasia (BPD), cerebral lesions (1, 2), among others (3, 4). Furthermore, each day of exposure to ampicillin or cephotaxime is associated with a 16% reduction in the anaerobe species in the intestinal microbiome in VLBW (5). Even 48 h of antibiotic exposition within the first days of life, significantly affects the diversity of microbiome with effects still detectable at one year of age (6).

Blood cultures remain the gold standard for the diagnosis of early-onset sepsis (EOS). Current recommendations support the collection of at least 1 mL of blood to optimize diagnostic yield. However, in VLBW infants, obtaining this volume can be challenging due to their limited circulating blood volume, extreme prematurity, and concerns about iatrogenic anemia and the potential need for transfusion. Despite these constraints, when this volume is obtained and processed using modern continuous-monitoring systems, blood cultures can detect up to 98% of bacteremia with bacterial loads ≥4 colony-forming units (CFU)/mL and approximately 95% of low-colony-count bacteremia (7–9).

Importantly, time-to-positivity studies have shown that most clinically significant pathogens are detected early during incubation. In particular, up to 98% of gram-negative organisms are identified within the first 24 h, supporting the reliability of blood cultures for early clinical decision-making (10–12).

The guidelines for the management of EOS in preterm infants recommend the discontinuation of antibiotics after 36–48 h of incubation (13). However, findings from recent studies suggest that in clinical practice, the exposure might be longer than current recommendations (2).

Antibiotic stewardship interventions targeting VLBW infants center on evaluating infants at low suspicion of EOS to determine whether antibiotics should be initiated (14). The application of contemporary blood culture techniques presents a valuable opportunity to implement an additional intervention point to reduce antibiotic overuse in this population and ensuring optimal discontinuation.

For over 10 years, our team has been working to reduce antibiotic exposure in VLBW infants. Our aim is to decrease the number of newborns who receive antibiotics at birth and to discontinue treatment as early as possible in those for whom it is indicated, due to the suspicion of EOS (15). It is noteworthy that, despite the growing number of publications on reducing antibiotic use in VLBW infants, there is still limited information on the actual duration of antibiotic exposure and the precise timing of discontinuation.

Taking this into consideration, the main objective of our study was to quantify early antibiotic exposure in VLBW infants, measured in hours of life from birth until the time of the stopping order, among those empirically treated for suspicion of EOS with a negative blood culture.

Secondary objectives included evaluating whether birth time, time of blood culture collection, day of the week, gestational age, or birth weight influence the duration of exposure; describing the number of antibiotic doses received and the timing of discontinuation per antibiotic; and analyzing temporal trends in antibiotic exposure over the study period.

## Methods

### Study design and settings

A retrospective cohort study of VLBW infants admitted to the neonatal intensive care unit (NICU) between March 1 2021 to December 31 2023. The NICU is a IIIC level unit, with nearly 800 admissions annually, about 100 of whom are VLBW.

We included all VLBW infants born at a tertiary-care maternity unit who were admitted to the NICU, received empirical antibiotic treatment for suspicion of EOS within the first 72 h of life, and did not have microbiologically confirmed EOS. Infants transferred from other facilities, those with EOS, those with incomplete medical records, those who died in the delivery room, and those who died within the first 72 h of life while receiving active antibiotic treatment at the time of death were excluded.

For antibiotic metrics and timing analysis, we included those infants that received antibiotics for suspicion of EOS between birth and 72 h of life with negative blood culture. Patients who, despite having a negative blood culture, received antibiotic treatment for another justified reason were excluded. One such reason was clinical sepsis. Clinical sepsis was defined as the presence of clinical signs and symptoms consistent with infection, together with supportive laboratory findings and/or clinical deterioration, leading to the initiation and continuation of antibiotic therapy despite negative blood cultures, in the absence of an alternative confirmed diagnosis.

In accordance with NICU policy, antibiotic treatment is not initiated for VLBW infants following birth under the following conditions: cesarean delivery, absence of chorioamnionitis, and no rupture of membranes prior to delivery (14). If a VLBW infant is considered at risk for EOS and antibiotics are started, at 36–48 h after initiation, if the blood culture is sterile, antibiotics are discontinued.

The microbiology laboratory utilizes automated continuous monitoring systems for blood cultures, enabling the neonatologists to consult at any time of the day if there is any growth in the blood culture. Furthermore, the laboratory notifies the clinical team of the positivity of the blood culture result.

### Measures

#### Antibiotic use and antibiotic timing

We collected antibiotics received for suspicion of early onset sepsis during the first 72 h of life (ampicillin, gentamycin, cefotaxime and meropenem) with negative blood culture. All antibiotics were administered intravenously.

The timing of antibiotic administration was meticulously documented. We recorded the precise time of antibiotic prescription, administration, stopping order and last dose administration, including the day, hour, and minute of the events. The time of blood culture reception at the laboratory was also collected.

We defined the following antibiotic exposure and timing variables:

- Number of doses administered (ND): number of doses administered of each antibiotic during the first 72 h of life.
- Hours of stopping order: hours of life between birth and the time of stopping order for each antibiotic.
- Negative culture time until suspension: number of hours from the start of blood culture incubation to antibiotic discontinuation.

We also collected dosing interval and total doses administered in milligrams. Days of therapy (DOT) was calculated by multiplying the number of antibiotic doses by the dosing interval and dividing by 24 h. For cases where there was a change in the interval during the treatment, DOT was calculated by number of calendar days exposed to the antibiotic.

#### Time determinants

In order to examine the role of time as a determinant in antibiotic exposure, the following variables were defined.

Turnicity definitions: Morning shift: from 8:00 to 15:00; afternoon shift: from 15:01 to 22:00, night shift: from 22:01 until 07:59, weekend: from Friday 15:01 until Monday 07:59.

#### Neonatal outcomes

EOS was defined as sepsis occurring in the first 72 h of life with positive blood culture. Late onset sepsis (LOS) was defined as sepsis occurring after the third day of life with positive blood culture; BPD was defined as requiring oxygen at 36 weeks' postmenstrual age or at discharge, whichever came first; retinopathy of prematurity (ROP) stage ≥ III or those that needed treatment; necrotizing enterocolitis (NEC) Bell's Stage ≥ II; brain injury as intraventricular hemorrhage (grade III or associated with hemorrhagic infarction).

### Data collection

We included perinatal and delivery characteristics such as gestational age, birth weight, intrauterine growth restriction (IUGR), Apgar score at 5 min, delivery room resuscitation and clinical risk index for babies (CRIB).

We recorded clinical treatment characteristics such as ventilation mode and vasopressor treatment when starting and stopping antibiotics to consider severity as a determinant in the decision to start or stop antibiotics.

The data and the evaluated measures were extracted from historic electronic medical records, variables regarding antibiotic use were extracted from electronic prescribing software IntelliSpace Critical Care and Anesthesia, Phillips® Andover, MA. An anonymus database was built with the data obtained.

### Statistical analysis

A descriptive analysis of clinical, perinatal, and antibiotic treatment variables was performed. Quantitative variables were described using median and interquartile range (P25–P75) or mean and standard deviation, according to their distribution. Categorical variables were expressed as absolute frequencies and percentages.

To identify factors associated with greater antibiotic exposure, a generalized linear model was used, with the total days of antibiotic therapy (total DOT) as the dependent variable. After evaluating potential covariates, the final model retained the following variables: initiation of antibiotics during the weekend, hours of life at which ampicillin was discontinued, and the number of hours between blood culture collection and the decision to discontinue gentamicin. A *p*-value <0.05 was considered statistically significant.

For the comparative analysis between study years, appropriate statistical tests were applied according to the nature of the variables: ANOVA or Kruskal–Wallis tests for quantitative variables, and chi-squared or Fisher's exact tests for categorical variables.

All analyses were performed using Stata 18 software.

### Ethical aspects

The study adheres to the tenets of the Helsinki Declaration. The project was reviewed and approved by Ethics Committees of the 12 de Octubre University Hospital (24/305) and have granted a waiver for parental informed consent.

## Results

**Figure 1:**
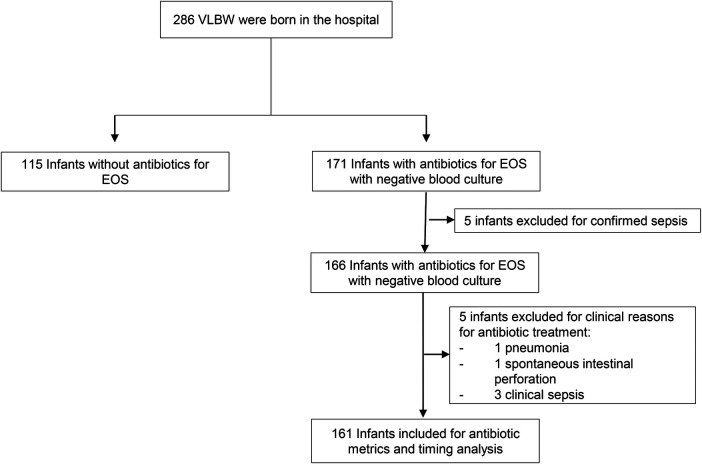
Flowchart of included VLBW admitted to the NICU, showing the selection process and final study population.

During the study period, 286 VLBW infants were admitted, and 166 met the inclusion criteria (Figure 1). However, some patients had justified reasons for antibiotic use; therefore, antibiotic use metrics were finally calculated for 161 patients.

The characteristics of the patients treated with antibiotics for suspicion of EOS, as well as their treatment and neonatal outcomes, are described in Table 1. Regarding neonatal management and outcomes, most infants required respiratory support at the initiation of antibiotic therapy, predominantly non-invasive ventilation. The proportion of infants requiring invasive ventilation or vasoactive support remained low. In terms of outcomes, bronchopulmonary dysplasia was observed in 18.5% of cases, retinopathy of prematurity in 9.9%, brain injury in 8.4%, and late-onset sepsis in 26% of infants (Table 1).

**Table 1:** Perinatal, demographic, clinical characteristics, treatment features, and neonatal outcomes of VLBW infants treated with antibiotics for suspicion of EOS (*N* = 161).

Variable	Infants characteristics
Gestational age. Median (PC 25- PC75)	27 (26 -29) weeks
Birth weight. Median (PC 25- PC75)	990 (800–1,262) g
Birth weight categorized	
≤750 g (%)	30 (18.6)
751-999 g (%)	51 (31.7)
1,000–1,249 g (%)	34 (21.1)
1,250–1,500 g (%)	46 (28.6)
Weight at discharge (*N* = 150). Median (PC 25- PC75)	2,470 (2,260–2,805) g
Antenatal corticosteroids (%)	154 (95.6)
Caesarean section (%)	95 (59)
Multiple birth (%)	60 (37.3)
Female (%)	81 (50.3)
IUGR (%)	32 (19.9)
Apgar 5 min	
0–4 (%)	16 (9.9)
5–7 (%)	39 (24.2)
8–10 (%)	106 (65.8)
CRIB. Median (PC 25- PC75)	3 (1–8)
Death (%)	11 (6.8)
Born during weekend (%)	71 (44.1)
Shift of admission	
Morning (%)	42 (26.1)
Afternoon (%)	58 (36.0)
Night (%)	61 (37.9)
NICU stay, days. Median (PC 25- PC75)	30 (13–51)
Total stay, days. Median (PC 25- PC75)	69 (47–96)
Neonatal treatment	
Surfactant treatment (%)	90 (56)
Days of invasive ventilation. Median (PC 25- PC75)	1 (0–6)
Mechanical ventilation at the start of antibiotics	
Non-Invasive mechanical ventilation (%)	104 (64.6)
Invasive mechanical ventilation (%)	41 (25.5)
HFOV (%)	16 (9.9)
Mechanical ventilation at the end of antibiotics	
No ventilatory support	3 (1.9)
Non-Invasive mechanical ventilation	127 (78.9)
Invasive mechanical ventilation	19 (11.8)
HFOV	12 (7.5)
FiO_2_ at the start of antibiotics. Median (PC 25- PC75)	0.21 (0.21–0.35)
FiO_2_ at the end of antibiotics. Median (PC 25- PC75)	0.21 (0.21–0.25)
Vasoactive drugs at the start of antibiotics	
1 vasoactive drug (%)	11 (6.8)
≥ 2 vasoactives drugs (%)	6 (3.7)
Any vasoactive drug and corticosteroid (%)	3 (1.9)
No vasoactive treatment (%)	141 (87.6)
Vasoactive drugs at the end of antibiotics	
1 vasoactive drug (%)	13 (8.1)
≥ 2 vasoactives drugs (%)	10 (6.2)
Any vasoactive drug and corticosteroid (%)	4 (2.5)
No vasoactive treatment (%)	134 (83.2)
Neonatal Outcomes	
ROP (%) (*N* = 151)	15 (9.9)
BPD (%) (*N* = 151)	28 (18.5)
Brain Injury (%) (*N* = 154)	13 (8.4)
Late Sepsis (%) (*N* = 154)	40 (26)

WK, weeks; g, grams; IUGR, intrauterine growth restriction; CRIB, clinical risk index for babies; NICU, neonatal intensive care unit; HFOV, high frequency oscillatory ventilation; FiO*_2_*, fraction of inspired oxygen; ROP, retinopathy of prematurity; BPD, bronchopulmonary dysplasia.

Regarding antibiotic treatment, of the total VLBW infants born during the study period, 59.8% (171/286) received antibiotics after birth, and 56.3% (161/286) received them empirically for infectious risk without later confirmation of sepsis or any other reason justifying antibiotic use. All patients received ampicillin, 99.4% (160/161) received gentamicin, and 6.2% (10/161) received cefotaxime. A total of 23% (37/161) of the patients received antibiotics for more than 48 h despite negative blood cultures. Table 2 describes the data on antibiotic use and exposure time in these patients. This table shows that, although the stopping order for antibiotics is issued before 48 h, it is still far from 24 h, both considering hours of life (45 h for ampicillin and gentamicin) and culture incubation time (42 h for ampicillin and gentamicin).

**Table 2:** Antibiotic metrics and timing variables for VLBW with antibiotic treatment for suspicion of EOS (*N* = 161).

Antibiotic metrics	Median (PC 25- PC75)
ND Ampicillin (*n* = 161)	3.94 (3–5)
ND Gentamicin (*n* = 160)	1.0 (1.0- 2.0)
ND Cefotaxime (*N* = 10)	3 (2.25–4)
DOT Ampicillin (*n* = 161)	2 (1.5–2.5)
DOT Gentamicin (*n* = 160)	2.00 (2.0–3.0)
DOT Cefotaxime (*N* = 10)	1.5 (1.12–2)
Antibiotic timing	
Hours of stopping order for Ampicillin	45.0 (38.0- 52.0)
Negative culture time until suspension for ampicillin	42.2 (10.7)
Hours of stopping order for gentamicin	45.0 (38.0–52.0)
Negative culture time until suspension for gentamicin	42.0 (35.0–48.0)
Hours of stopping order for cefotaxime	63.0 (30.0)
Negative culture time until suspension for cefotaxime	54.9 (30.8)

ND, number of dosis; DOT, days of treatment.

With respect to factors associated with greater antibiotic exposure, measured as days of antibiotic therapy (DOT), in patients without confirmed sepsis, the generalized linear model identified that initiating antibiotic treatment during the weekend increased exposure by 0.46 days [adjusted coefficient = 0.455; 95% CI (0.148, 0.762); *p* = 0.004] (Table 3).

**Table 3:** Multivariate generalized linear model of factors associated with total DOT in VLBW infants.

Multivariate analysis: generalized lineal model				
Outcome: total DOT	Adjusted coefficient	Standard error	CI 95%[ ]	*p*
Starting antibiotic on weekend	0.455	0.157	[0.148, 0.762]	0.004
Hours of life when stopping ampicillin order	0.040	0.008	[0.024, 0.055]	<0.0001
Incubation time for stopping Gentamicin order	0.053	0.007	[0.040, 0.066]	<0.0001
Obs = 155; Residual df = 151; Deviance = 141.760; AIC = 2.800; BIC = −619.797				

DOT, days of treatment.

When comparing results over time, the number of doses of ampicillin administered decreased in 2023 compared with the two previous years. For gentamicin, no difference was found. Regarding the time from birth to antibiotic discontinuation, there was a statistically significant decrease from 2021 to 2023 for both gentamicin and ampicillin. The results of antibiotic exposure according to the study years are shown in Table 4.

**Table 4:** Comparison of antibiotic exposure and administration patterns by study year.

Study year	2021	2022	2023	P*
DOT Ampicillin	2.1 (0.5)	2.0 (0.5)	1.8 (0.4)	0.045
DOT Gentamicin	2.0 (2.0–4.0)	2.0 (2.0–3.0)	2.0 (2.0–3.0)	0.578
DOT Cefotaxime	2.3 (1.2)	1.0**	1.5 (0.5–2.0)	0.413
Total, DOT	4.0 (4.0–6.0)	4.0 (3.5–5.0)	4.0 (3.5–5.0)	0.138
Total, dosis Ampicillin	4.0 (4.0–5.0)	4.0 (1.1)	3.7 (0.9)	0.029
Total, dosis Gentamicin	1.0 (1.0–2.0)	1.0 (1.0–2.0)	1.0 (1.0–2.0)	0.781
Total, dosis Cefotaxime	4.5 (2.4)	2.0**	3.2 (2.5)	0.938
Hours of life when stopping ampicillin order	47.0 (43.0–54.0)	44.0 (39.0–55.0)	43.0 (38.0–49.0)	0.023
Hours of life when stopping gentamicin order	47.0 (42.0–54.0)	44.0 (39.0–54.0)	42.0(36.0–48.0)	0.009

Values are presented as mean (SD) or median (IQR), as appropriate *Kruskar Wallis/ANOVA test for cuantitative variables/Chi 2 or Fisher test for categorical variables; **only one case.

## Discussion

This study provides a detailed analysis of antibiotic exposure in VLBW infants who were empirically treated for suspected EOS but had no microbiological evidence of infection. The primary objective was to accurately quantify antibiotic exposure in this group of infants. In this regard, the results show that the median time from birth to the stopping order was 45 h for both ampicillin and gentamicin, and when considering blood culture incubation time, 42 h for both antibiotics. This finding is consistent with current clinical practice and aligns with existing guideline recommendations, which support discontinuation of antibiotics at 36–48 h in the absence of microbiological confirmation. Notably, evidence from time-to-positivity studies indicates that most clinically significant pathogens are detected within the first 24 h of incubation, particularly when optimal blood culture conditions are achieved (11, 16). In this context, our results suggest that antibiotic exposure in routine practice may still extend beyond the earliest time points at which infection can be reasonably excluded. As a positive aspect, we can highlight that over the 3 years analyzed, the stopping order has been issued earlier, and in 2023 ampicillin was stopped at 43 h of life and gentamicin at 42 h.

We considered this study to be highly relevant because, although there is a trend toward earlier discontinuation of antibiotics, our team perceived that treatment was being stopped around 36 h, but the results show that we are still far from that goal. Knowing these results has encouraged all neonatologists to be more aware and to avoid delaying the discontinuation order, recognizing, as is well known, that every administered antibiotic dose counts, both for the clinical evolution of the infant and for the development of antimicrobial resistance.

Studies that have implemented an automatic stopping order at 48 h after the start of treatment did not achieve results very different from ours. The first, published in 2019, included all admitted newborns who were given antibiotics for any reason (only around 10% were VLBW infants). After implementing an automatic stop order, the overall population of newborns received 3 doses of ampicillin and 1.3 doses of gentamicin (17). In our case, in 2023, 3.7 doses of ampicillin and 1 dose of gentamicin were administered. In the second study, from 2023, an automatic stop order at 48 h was also applied, and the study population included all newborns receiving antibiotics. Based on the data reported by the authors, the final antibiotic exposure was 3.7 days (18). According to these data, an automatic 48-hour antibiotic stop order does not shorten exposure time more than our team has achieved.

One particularly relevant finding of our study was that being born during the weekend was associated with an increase of 0.55 days in antibiotic treatment compared to being born on a weekday (*p* = 0.001). This finding raises important questions about the influence of logistical or organisational factors on clinical decision-making. It is possible that there is a greater tendency to maintain empirical treatments as a precaution during weekends because neonatal units are typically staffed only by on-call neonatologists during these periods, resulting in an increased workload. While understandable, this pattern may have important clinical implications. As previously mentioned, several studies have shown that in VLBW infants, each additional day of antibiotic therapy increases the risk of bronchopulmonary dysplasia, necrotizing enterocolitis, and even mortality (2, 19, 20).

A strength of this study is that it was conducted after previous work to limit antibiotic prescription for EOS risk in VLBW infants, and only 58% of these infants received antibiotics for EOS risk during the study period (15). Therefore, our study focuses on an already selected population of VLBW infants for whom there was a prior assessment of actual EOS risk, and antibiotics were prescribed accordingly. To date, there is very limited information available on the duration of antibiotic exposure in this specific group of selected infants with a higher suspicion of EOS. Another strength is the meticulous documentation of the timing of each event related to antibiotic administration, which allowed us to accurately analyze factors associated with exposure duration. Additionally, the analysis was performed on a large cohort of VLBW infants. As a limitation, this study is retrospective and single-center.

Although previous efforts in our neonatal unit to limit antibiotic exposure in VLBW infants have had positive results, we are still discontinuing antibiotics closer to 48 h than to 36 h of life. We believe it is important for teams to know the actual timing of antibiotic discontinuation, as perception often does not match reality. Being born on weekends is associated with increased antibiotic exposure, which could be avoided by prioritizing discontinuation orders over other tasks. Many VLBW infants no longer receive early antibiotics, so having precise information about the group of VLBW infants in whom EOS risk was considered will allow us to further shorten exposure time in this higher-risk population.

## Data Availability

The raw data supporting the conclusions of this article will be made available by the authors, without undue reservation.
