# Correction: Duration of early empirical antibiotic exposure in very low birth weight infants with suspected early-onset sepsis and associated factors: a retrospective cohort study

**DOI:** 10.3389/fped.2026.1931937

**Published:** 2026-07-27

**Authors:** Catalina Morales-Betancourt, Maria Dolores Canales-Siguero, Marta Fernández-Gaitán, Adriana Montealegre-Pomar, Elena Bergon-Sedín, Concepción De Alba-Romero, Carmen Rosa Pallás-Alonso, Maria Teresa Moral-Pumarega

**Affiliations:** 1 Research Institute Hospital 12 de Octubre, Madrid, Spain; 2 Departament of Neonatology, Hospital Universitario 12 de Octubre, Madrid, Spain; 3 Escuela de Doctorado, Universidad de Alcalá, Ciencias de la Salud, Madrid, Spain; 4 Departament of Pharmacy, Hospital Universitario 12 de Octubre, Madrid, Spain; 5 Hospital Universitario San Ignacio, Bogotá, Colombia

**Keywords:** antibiotic exposure, antibiotic stewardship, early-onset sepsis, sepsis, very low birth weight infant

**Error in figure/table**

Wrong content

There was a mistake in the content of table 4, and a mistake in numbering the tables as published.

The Table 4 included in the published article does not correspond to the results described in the manuscript. Table 4 should be renumbered as Table 3 and positioned before the currently published Table 3.

The corrected table with the corrected number appears below.

**Table 3:** Multivariate generalized linear model of factors associated with total DOT in VLBW infants.

Multivariate analysis: generalized lineal model				
Outcome: total DOT	Adjusted coefficient	Standard error	CI 95%[ ]	*p*
Starting antibiotic on weekend	0.455	0.157	[0.148, 0.762]	0.004
Hours of life when stopping ampicillin order	0.040	0.008	[0.024, 0.055]	<0.0001
Incubation time for stopping Gentamicin order	0.053	0.007	[0.040, 0.066]	<0.0001
Obs = 155; Residual df = 151; Deviance = 141.760; AIC = 2.800; BIC = −619.797				

DOT, days of treatment.

The original version of this article has been updated.

